# Mechanical performance of sustainable concrete containing industrial waste admixtures of SF, FA, CCI, and crushed quartz at different water–binder ratios

**DOI:** 10.1038/s41598-025-34612-0

**Published:** 2026-01-22

**Authors:** Seleem S. E. Ahmad, Abdo-Alfatah A. A. Graf, Mohamed Khalifa Bneni

**Affiliations:** 1 Faculty of Engineering, Zagazig University, Zagazig, 44519 Egypt; 2 Faculty of Engineering, University of Sebrata, Regdalin, 16418 Libya; 3 Faculty of Engineering, University of Zawia, Zawia, 16418 Libya

**Keywords:** Industrial wastes, Mineral admixtures, Quartz powder, Compressive strength, Sustainability, Engineering, Environmental sciences, Materials science

## Abstract

The present study examined the mechanical performance of sustainable concrete with silica fume (SF), fly ash (FA), cooled cast iron (CCI), and crushed quartz powder (CQ) used as partial cement replacements at three water-to-binder ratios (0.36, 0.41, and 0.46). Fifteen concrete mixtures were cast and tested for compressive, splitting tensile, and flexural strengths at 7, 28, and 90 days. Results indicated that the lowest water–binder ratio of 0.36 produced the highest strengths, consistent with values of 78 MPa compressive strength at 28 days and 87.8 MPa at 90 days, which were better than those obtained from mixes having the other two ratios by up to 34% in early ages and by up to 33% in later ages. The partial replacement of SF by FA lowered the early-age strength, but very significant long-term gains were recorded; replacing three-quarters of SF with FA increased the 90-day compressive strength by 5.9%, 30.4%, and 30.1% for groups 0.36, 0.41, and 0.46, respectively, compared to their corresponding mixes with SF only. Incorporating CQ led to reductions in strength at 28 days of 9.3–13.4%; however, reductions at 90 days remained modest, 8.1–13.6%, indicating improved long-term pozzolanic contributions. It is noted that tensile-to-compressive strength ratios for all mixtures ranged from 9.8 to 12.2%, and flexural-to-compressive ratios ranged from 13.6 to 14.7%, which are better than the ACI318 predictive correlations for concretes using industrial by-products. The results confirm that optimized SCM blending higher fly-ash proportions at lower water–binder ratios enhances long-term mechanical performance while enabling significant cement reduction, supporting more environmentally sustainable high-strength concrete.

## Introduction

The construction sector, especially cement production, is a major contributor to carbon emissions from human activities^1^. Therefore, researchers aim to contribute to the decarbonization of the construction industry by partially replacing cement with lower-energy, low-carbon alternatives, such as industrial waste and mineral additives^2^. It should be noted that replacing cement directly reduces the embodied energy and carbon footprint of a volumetric unit of concrete. Integrating industrial waste into concrete production has emerged as a crucial strategy to enhance the sustainability and performance of construction materials. Within the framework of the circular economy, there is an urgent need to valorise large volumes of industrial waste that are often disposed of in landfills. Incorporating this waste into concrete designs not only improves properties but also reduces landfill space requirements^3,4^. The contribution of low-carbon materials is completed only by proving their reliability and durability comparable to or superior to conventional concrete. So, the study of mechanical properties in the short and medium term serves a deeper goal of determining the expected service life^5^. Using sustainable concrete has become increasingly important for improving the performance and sustainability of modern construction practices, especially concerning its resistance to permeability and harmful chemical reactions^6^.

Fly ash, silica fume, metakaolin, and ground granulated blast furnace slag are promising materials for replacing Portland cement. The investigations indicated that GGBS improves concrete’s mechanical properties and durability, especially at later ages, depending on the replacement amount. According to the results, the compressive strength of the concrete mix is increased by about 17% when 20% waste foundry sand and 5% silica fume are added. Furthermore, this optimization rises to 23% when 10% metakaolin is added^7–9^. Lathe iron waste particulates were used as a partial substitute for fine aggregate at different weight percentages (5%, 10%, 15%, and 20%) to fabricate sustainable concrete. The tests were conducted on mixtures, including workability, ultrasonic pulse velocity, compressive strength, splitting tensile strength, and flexural strength, to investigate the properties of the alternative concrete compared with conventional concrete. It remarkably enhanced the tensile, flexural, and compressive strength of the concrete by up to 13%, 19%, and 38%, respectively^10^.

Paper sludge ash is a significant byproduct of the paper manufacturing industry. It uses sludge ash powder as a partial substitute for cement in concrete and a partial or full replacement for natural sand with waste glass aggregate. The results revealed that integrating paper sludge ash can improve compressive strength at specific replacement levels, consistent with prior research on the pozzolanic activity of such waste materials^11^. Another study incorporates waste fly ash, waste perlite powder, and recycled cellular concrete as partial substitutes for cement and aggregate in concrete mix design. The waste fly ash and waste perlite powder can replace up to 15% of cement, while the recycled cellular concrete should not exceed 15% due to performance concerns. Accordingly, the degree of reaction of waste perlite powder at 56 days was 19.53% ^12^. Over the last two decades, many investigators have focused on decreasing environmental effects by replacing basic materials with recycled aggregate in concrete^13–17^.

Wood waste is an important industrial waste that has been studied by many researchers to enhance the mechanical performance of sustainable concrete containing wood ash. Increase compressive strength and split tensile strength of specimens up to 20% substitution of wooden ash compared to reference concrete, due to pozzolanic reaction and micro filler of wooden ash^18^. Furthermore, several studies reviewed the production and development of sustainable concrete using different industrial waste^19,20^. Many studies focused on analysing the properties of concrete incorporating waste glass as a filling material in proportions of 5%, 10%, 15%, 20%, 25%, and 30% by weight of cement. The results demonstrate that the addition of glass waste reduces the workability of concrete; in contrast, mechanical performance was increased up to a 20% substitution of waste glass and then gradually decreased^21–24^. On the other hand, the effect of snail shell powder and quartz powder as partial substitutes for cement in order to lessen the environmental impact of concrete was examined^25^. The study assessed the workability and mechanical qualities of snail shell powder and quartz powder at 2.5–10% concentrations as supplemental cementitious materials in the construction of sustainable concrete. The sustainable concrete containing 7.5% snail shell powder exhibits compressive, tensile, and flexural strengths of 125%, 120%, and 112% of the control concrete, respectively. These values exceed those of concrete containing 7.5% quartz powder as a partial cement.

This research examines the influence of different water-to-binder ratios on the mechanical properties of sustainable concrete by evaluating three specific ratios (0.36, 0.41, and 0.46) and assessing compressive, tensile, and flexural strengths at 7, 28, and 90-day intervals. It seeks to ascertain the impact of substituting silica fume with fly ash at proportions of 25%, 75%, and 100% on strength development at these intervals. The study also examines how crushed quartz powder can be used as an additional cementitious material and how it affects strength performance, especially when mixed with silica fume and fly ash, across a range of water-to-binder ratios and curing times. The goals include identifying real-world links between tensile-to-compressive and flexural-to-compressive strength in sustainable concrete made from industrial by-products, and then comparing the results with the predictive models in ACI 318. The study also uses Scanning Electron Microscopy (SEM) to examine the microstructural features of certain combinations and link morphological attributes to mechanical performance.

## Experimental work

### Justification for mix proportioning in the experimental design

The experimental program outlined in Table 1 focuses on evaluating concrete mix designs with varying compositions of cementitious materials, supplementary cementitious materials (SCMs), and additives. The goal appears to be optimized strength and performance through systematic adjustments to component ratios and material substitutions. Each mix is identified by a unique Mix ID (e.g., M1S1P1, M2S3Q1P1), where: M1, M2, and M3 denote the primary mix group, differing in water-to-(cement + SCMs) ratio (W/B) (0.36, 0.41, 0.46). SF and QP indicate the inclusion of silica fume (SF), fly ash (FA), or crushed quartz powder (QP). Numerical suffixes (e.g., S1, F1, Q1) specify proportions or combinations of SCMs. Cement content increases across groups (e.g., 375 kg in M1S1P1, 425 kg in `M3S1P3) as the W/B ratio rises (0.36 → 0.46). Silica fume (SF) is a primary SCM in early mixes (e.g., 125 kg in M1S1P1), but its quantity decreases in later groups (e.g., 75 kg in M3S1P3). Fly Ash (FA) and Crushed Quartz Powder (QP) are often substituted for silica fume (e.g., M1S2F1P1 uses 31 kg FA Combined SCMs: Some mixes (e.g., M1S4F3Q1P1) blend SF, FA, and QP in equal or proportional amounts to study synergistic effects. This structured experimental approach allows systematic analysis of how material substitutions and ratio adjustments influence concrete performance, supporting tailored mix designs for specific engineering requirements.

**Table 1:** Mix propositions and outline of the experimental program.

Mix ID	W/B	Cement, C (kg)	Fine aggregate (kg)	Silica fume, SF (kg)	Fly ash, FA (kg)	Crushed quartz powder, QP (kg)	Cooler cast iron, CCI (kg)	Viscocret 3425 (kg)
M1S1P1	0.36	375	1700	125	–	–	12.5	2.5
M1S2F1P1				94	31	–		5.0
M1S3F2P1				31	94	–		7.5
M1S4F3Q1P1				47	47	31		7.5
M1F2Q1P1				–	94	31		10.0
M2S1P2	0.41	400	1700	100	–	–	10	2.5
M2S2F1P2				75	25	–		5.0
M2S3F2P2				25	75	–		7.5
M2S4F3Q1P2				37.5	37.5	25		7.5
M2F2Q1P2				–	75	25		10.0
M3S1P3	0.46	425	1700	75	–	–	7.5	2.5
M3S2F1P3				56.25	18.75	–		5.0
M3S3F2P3				18.75	56.25	–		7.5
M3S4F3Q1P3				28.12	28.12	18.75		7.5
M3F2Q1P3				–	56.25	18.75		10.0

### Material properties

**Fig. 1:**
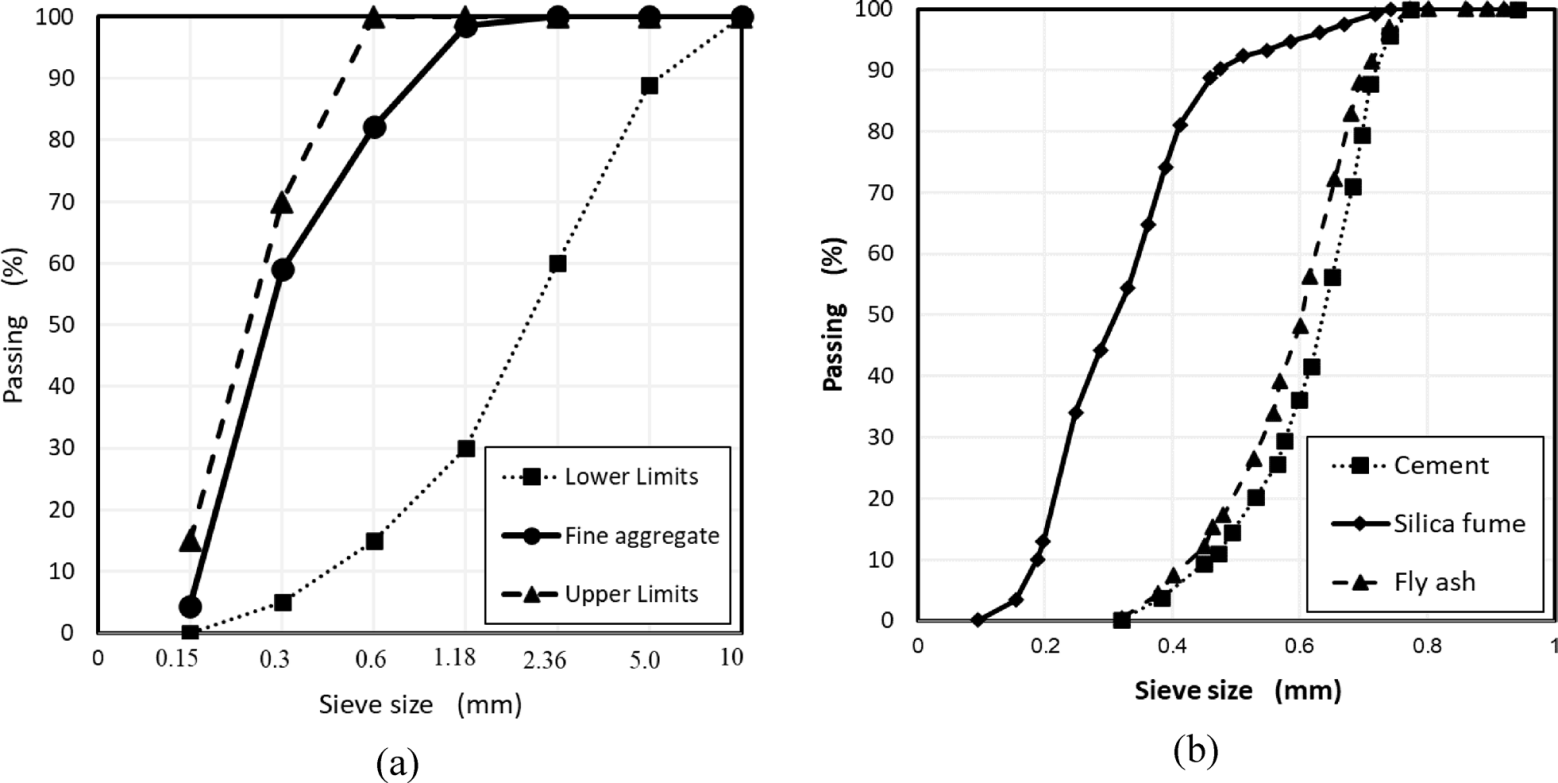
(**a**) Grading curve of the used sand, (**b**) Particle Size Distribution of Cement, Silica fume and Fly ash.

Ordinary Portland Cement (OPC), produced by one of the local companies Sina Company, was used in all mixes. The grade of the used Portland Cement was CEM I 52.5 N, which complies with BS EN 197-1/2011^26^ and Egyptian Standard Specification ESS 4756-1/2009^27^. Tables 2 and 3 show the chemical analyses and physical properties of the used cement supplied by the manufacturer and the limits according to the Egyptian Standard Specification ESS 2421/2009^28^. The fine aggregate used in this investigation was natural siliceous sand. Table 4 shows the physical properties of the used sand. Sand size particle distribution was determined by sieve analysis according to ESS 1109/2002^29^, and fineness modulus, and specific gravity of 2.65 and 2.63, respectively. The grading curve of the used sand and the upper and lower limits according to ESS^30^ are shown in Fig. 1a. Tap water was used for casting and curing specimens free from deleterious material.

**Table 2:** Chemical analyses of used OPC.

Oxides	SiO_2_	Al_2_O_3_	Fe_2_O_3_	CaO	MgO	SO_3_	Na_2_O	K_2_O	L.O. I
Results %	21.2	4.67	5.05	64.73	1.5	2.05	0.3	0.22	2.6

**Table 3:** Physical properties of the used cement (52.5 N).

Properties	Test result	Limits*
Specific surface area (Blain) (m^2^/kg)	350	Not less than 275
Specific weight	3.15	–
Initial setting time (min)	80	Not less than 45 min
Final setting time (h)	4	Not more than 10 h

ESS 4756-1/2009.

**Table 4:** Physical properties of the used silica fume and fly ash.

Property	Results	
	Silica fume	Fly ash
Specific surface area (m^2^/kg)	17.8 × 10^3^	356
Particle size (µm)	7.00	7.00
Bulk density (kg/m^3^)	300	320
Specific gravity	2.25	2.20
Color	Gray powder	Grey powder

**Fig. 2:**
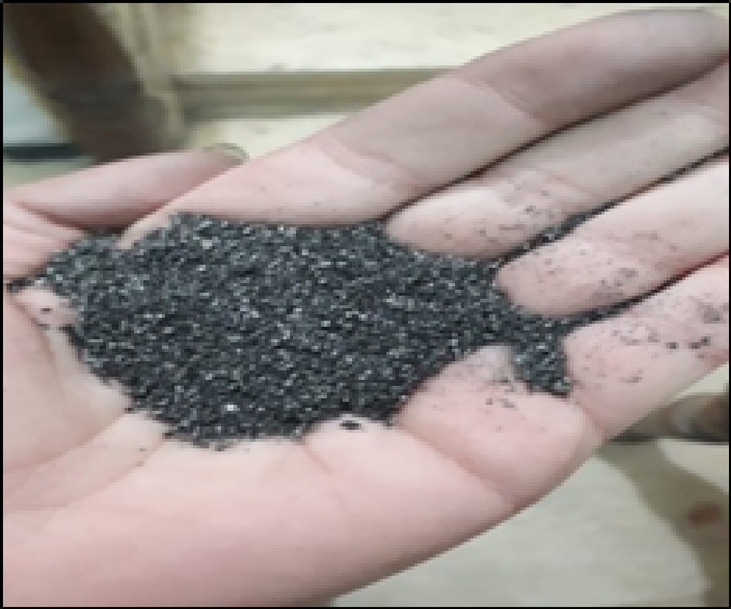
Cooler cast iron sample.

**Fig. 3:**
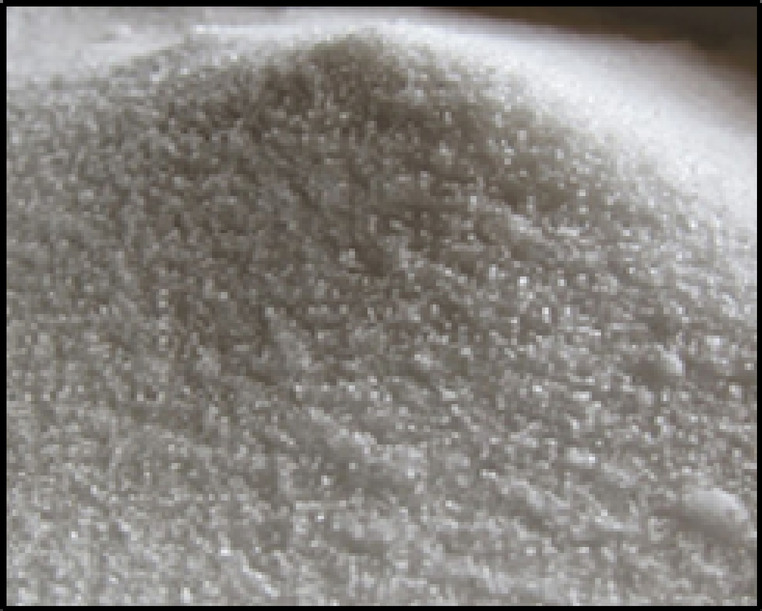
ACC crushed quartz powder.

The superplasticizer was Sika ViscoCrete-3425, which complies with ASTM C-494 types G and F and BS EN934 part 2:2001. It was a kind of high-range water reducer (HRWR) of third-generation superplasticizer for homogenous concrete and mortar as an aqueous solution of modified polycarboxylates ether-based superplasticizer from Sika Company. Dosages of this superplasticizer were varied in each sample to obtain similar consistency. Silica fume (SF) from Sika Egypt Company, which complies with ASTM C-1240, was partially replaced with cement in 25% and 18.75%. Fly Ash brought from Sika Egypt Company, which complies with ASTM C-618-12a, was used as a partial replacement of cement by 25% and 18.75%. The physical properties and chemical composition of the used SF and FA are given in Tables 4 and 5, respectively. The cooled cast iron used was brought from local lathes in Zagazig, as shown in Fig. 2. The chemical composition of the CCI used is supplied by the international accreditation service with ASTM C-114-00 and ASTM114-18. Crushed quartz powder, QP, was brought from ACC Company in Tanta, as shown in Fig. 3. The chemical analysis using X-ray spectroscopy (XRF) at room temperature is given in Table 5 for CCI and ACC.

**Table 5:** Chemical analyses of used SF, FA, CCI and CQ.

Oxides	SiO_2_	Al_2_O_3_	Fe_2_O_3_	CaO	MgO	SO_3_	Na_2_O	K_2_O	H_2_O	L.O.I
SF	96.00	1.10	1.45	1.20	0.18	0.25	0.45	1.20	0.85	–
FA	36.10	25.03	8.66	3.10	1.24	0.59	0	1.08	0.91	–
CCI	3.45	0.91	92.80	0.44	0.15	0.23	0.25	0.05	–	0.10
CQ	99.3	0.33	0.027	0.01	0.08	0.023	0.01	0.21	–	0.05

### Mixing, casting, and curing of the mixtures

**Fig. 4:**
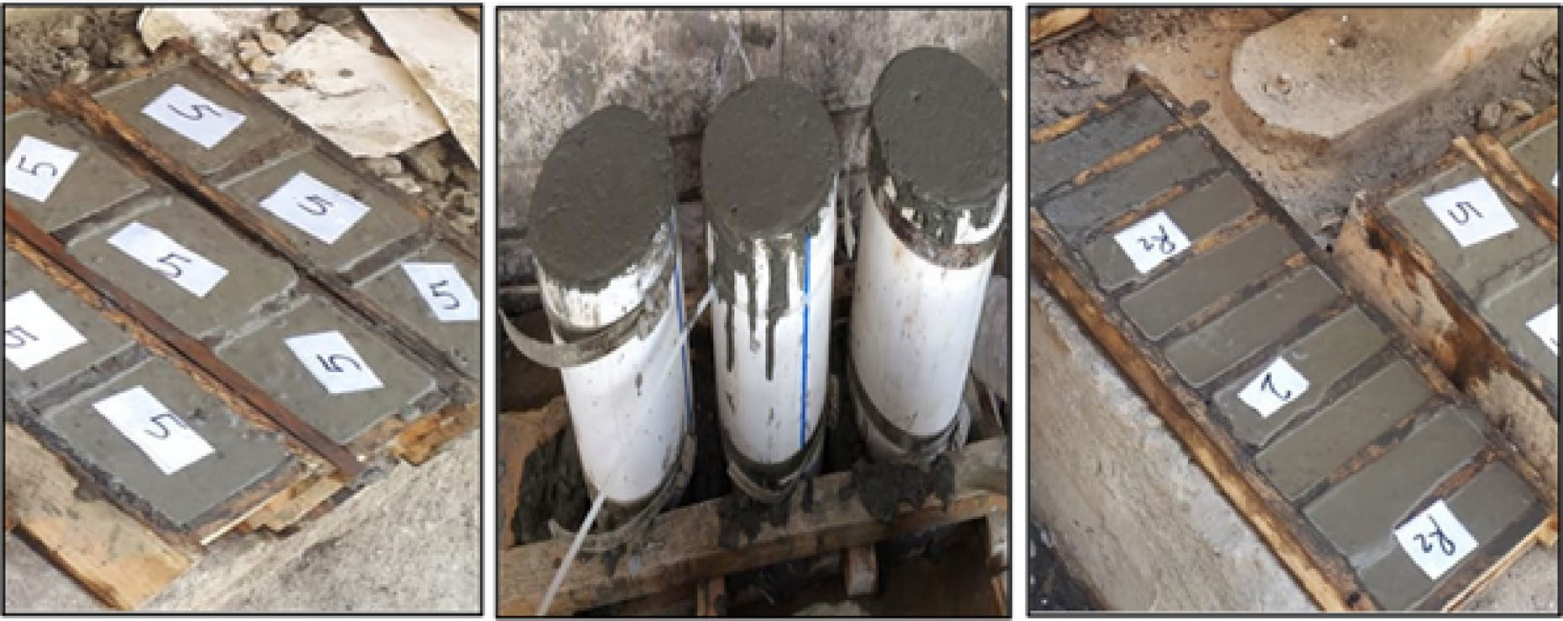
Cubic, cylindrical, and prism specimens in the wooden molds.

**Fig. 5:**
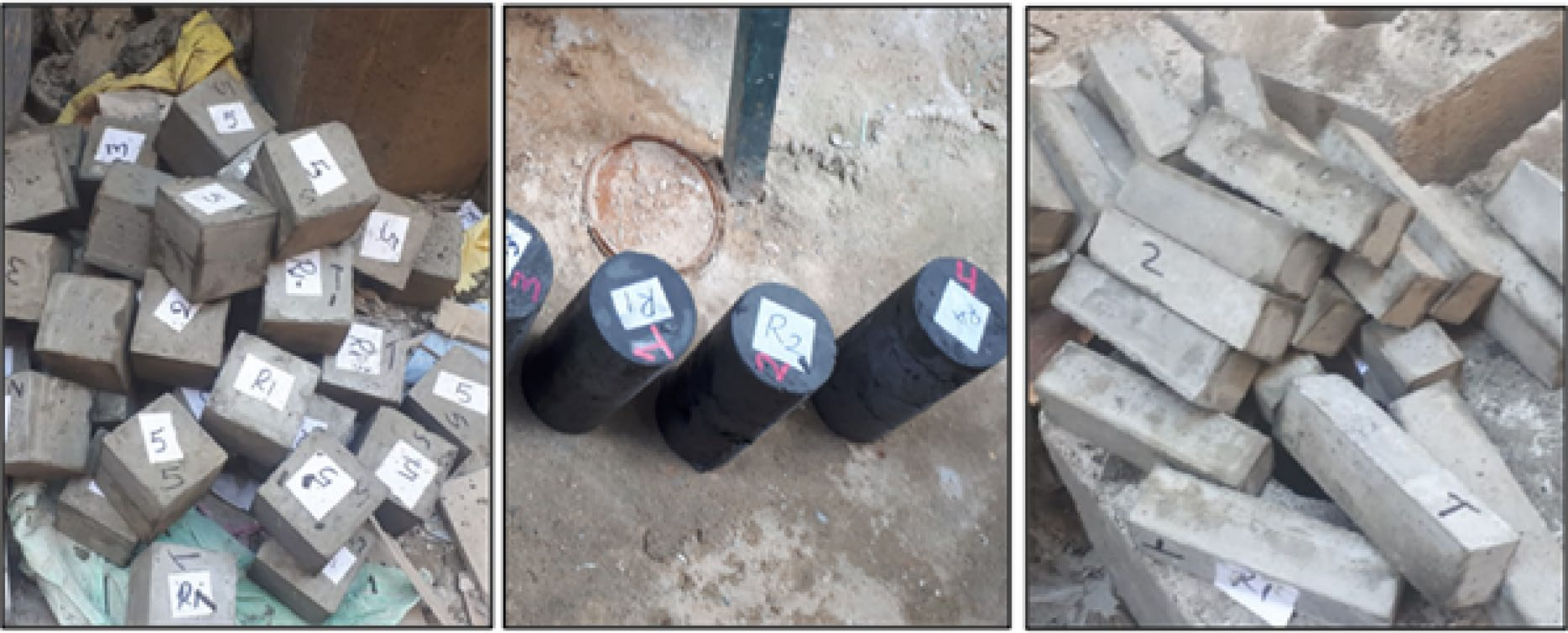
Cubic, cylindrical, and prism specimens after losing the wooden molds.

**Fig. 6:**
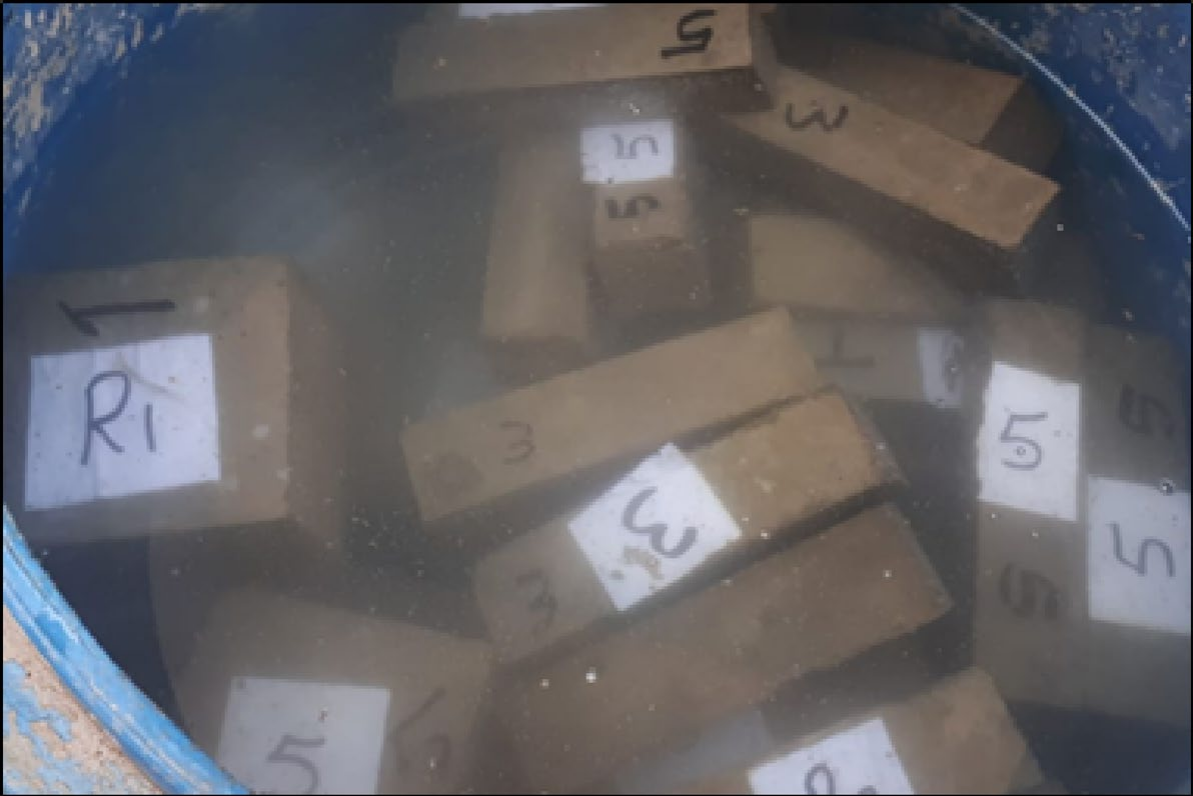
Curing of tested specimens.

The mixing procedures involved placing fine materials (cement-silica fume) in the mixer and mixing for 30 s. Then, add 3/4 of the water to the pan, mix gradually, and continue mixing for another 1 min. After that, add the required dosage of the superplasticizer to 1/4 of the necessary amount of water and mix for 30 s at high speed. Next, add the fine aggregate gradually, followed by the cooled cast iron, and mix for another 2 min. The fresh concrete was cast in 100 mm size cubes for compressive tests (three cubes for each mix) and 40 × 40 × 16 mm prismatic shapes for compressive and flexural tests, as shown in Figs. 4 and 5. After pouring the mixes into oiled molds, the electric vibrator was used to ensure good compaction. Then, the specimens were surface-smoothed and covered with wet hessian. All specimens were remolded 1 day after casting and cured in a standard water tank for 7, 28, and 90 days. The hardened concrete samples were output from the curing tank at the specified testing age as shown in Fig. 6.

### Hardened concrete testing

**Fig. 7:**
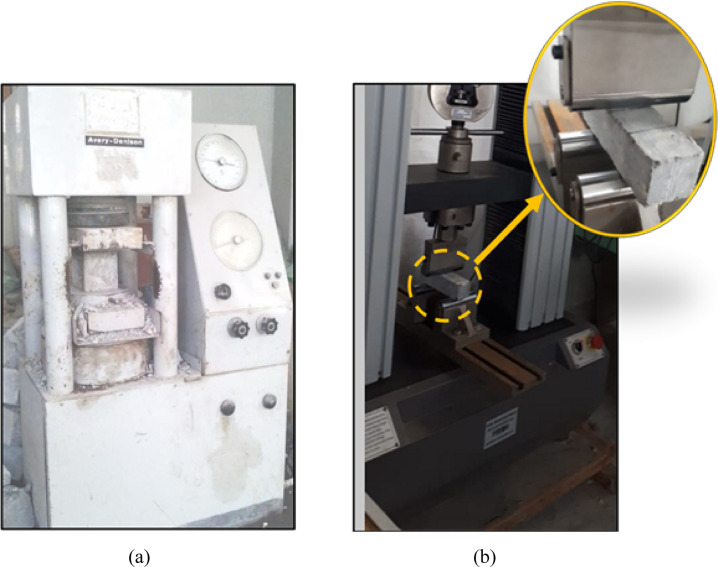
Test setup: (**a**) Compression test and (**b**) Flexural test.

The compression test was carried out on concrete to determine its compressive strength. After the specified curing duration, three cubes from each concrete mix were tested according to BS 1881-116^31^. This involved applying a compressive axial load to the cubes at a controlled rate until failure occurred. A compression testing machine with a maximum capacity of 2500 kN, as shown in Fig. 7a. A flexural test using center point loading was carried out on concrete as shown in Fig. 7b, to determine the flexural strength of the concrete. After the specified curing duration, three prisms from each concrete mix are tested according to ASTM C293^32^.

## Results and discussion

This section will investigate and discuss the results of mixtures depending on the parametric factors. Table 6 summarizes the experimental results of compressive, tensile, and flexural strengths for 7, 28, and 90 days. As can be noticed from Table 7, the relationship between $f_{t}$ and $f_{b}$ to $f_{c}^{\prime }$ ratios of sustainable concrete with various W/B ratios and statistical analysis.

**Table 6:** Compressive, tensile, and flexural strength results for all tested mixes at 7, 28, and 90 days.

Mix ID	W/B	7 -days			28 -days			90 -days		
		$f_{c}^{\prime }$(MPa)	$f_{t}$(MPa)	$f_{b}$(MPa)	$f_{c}^{\prime }$(MPa)	$f_{t}$(MPa)	$f_{b}$(MPa)	$f_{c}^{\prime }$(MPa)	$f_{t}$(MPa)	$f_{b}$(MPa)
M1S1P1	0.36	55.7	6.6	8	78	9.5	11.5	87.8	10.5	12.7
M1S2F1P1		48.8	5	7.2	70	6	10	87	11.9	13
M1S3F2P1		54.3	6.69	8.84	75	8	11	93	11.1	14.8
M1S4F3Q1P1		49	4.8	6.5	71	6.7	9.2	84.8	10.4	12.4
M1F2Q1P1		51.3	5.5	7	68	6.1	8.5	85.4	8.9	11.55
M2S1P2	0.41	51	5.03	6.98	67	6.7	9.3	82.6	8.04	11.15
M2S2F1P2		46.5	4.58	6.6	62	6.1	8.9	90.4	9.22	10.65
M2S3F2P2		50.2	5.1	6.7	67	6.9	9.1	87.4	10.3	11.9
M2S4F3Q1P2		45.45	4.55	5.8	61	6.2	8.3	79.2	9.4	10.45
M2F2Q1P2		43	4.17	5.2	58	5.7	7.1	75.5	7.84	9.1
M3S1P3	0.46	42.05	5.1	5.7	58	5.7	7.9	66.1	7.48	9
M3S2F1P3		36.25	3.5	4.78	50	5	6.6	67	7.7	8.52
M3S3F2P3		38.8	4.8	5.1	55	5.9	7.9	71.7	9.6	9.9
M3S4F3Q1P3		37.5	3.64	4.8	52	5.3	6.7	68.2	7.64	7.84
M3F2Q1P3		35.5	3.25	4.4	49	4.9	6.1	64.86	7.58	7.95

**Table 7:** Summary relationship between $f_{t}$ and $f_{b}$ to $f_{c}^{\prime }$ ratios of sustainable concrete with varies W/B ratios.

Mix ID	$f_{t} /f_{c}^{\prime }$(%)			$f_{b} /f_{c}^{\prime }$(%)			Mean $f_{t} /f_{c}^{\prime }$ (%)	SD $f_{t} /f_{c}^{\prime }$	Mean $f_{t} /f_{c}^{\prime }$ (%)	SD $f_{t} /f_{c}^{\prime }$
	7 -days	28 -days	90-days	7 -days	28 -days	90-days				
M1S1P1	11.85	12.18	11.96	14.36	14.74	14.46	12.00	0.002	14.52	0.002
M1S2F1P1	10.25	8.57	13.68	14.75	14.29	14.94	10.83	0.026	14.66	0.003
M1S3F2P1	12.32	10.67	11.94	16.28	14.67	15.91	11.64	0.009	13.62	0.008
M1S4F3Q1P1	9.80	9.44	12.26	13.27	12.96	14.62	10.50	0.015	13.62	0.009
M1F2Q1P1	10.72	8.97	10.42	13.65	12.50	13.52	10.04	0.009	13.22	0.006
M2S1P2	9.86	10.00	9.73	13.69	13.88	13.50	9.87	0.001	13.69	0.002
M2S2F1P2	9.85	9.84	10.20	14.19	14.35	11.78	9.96	0.002	13.44	0.014
M2S3F2P2	10.16	10.30	11.78	13.35	13.58	13.62	10.75	0.009	13.51	0.001
M2S4F3Q1P2	10.01	10.16	11.87	12.76	13.61	13.19	10.68	0.010	13.19	0.004
M2F2Q1P2	9.70	9.83	10.38	12.09	12.24	12.05	9.97	0.004	12.13	0.001
M3S1P3	12.13	9.83	11.32	13.56	13.62	13.62	11.09	0.012	13.60	0.000
M3S2F1P3	9.66	10.00	11.49	13.19	13.20	12.72	10.38	0.010	13.03	0.003
M3S3F2P3	12.37	10.73	13.39	13.14	14.36	13.81	12.16	0.013	13.77	0.006
M3S4F3Q1P3	9.71	10.19	11.20	12.80	12.88	11.50	10.37	0.008	12.39	0.008
M3F2Q1P3	9.15	10.00	11.69	12.39	12.45	12.26	10.28	0.013	12.37	0.001

$f_{t}$, Spitting tensile strength; $f_{b}$, Flexural strength; $f_{c}^{\prime }$, Compression strength; SD, Standard deviation.

### Effect of water–binder ratio

**Fig. 8:**
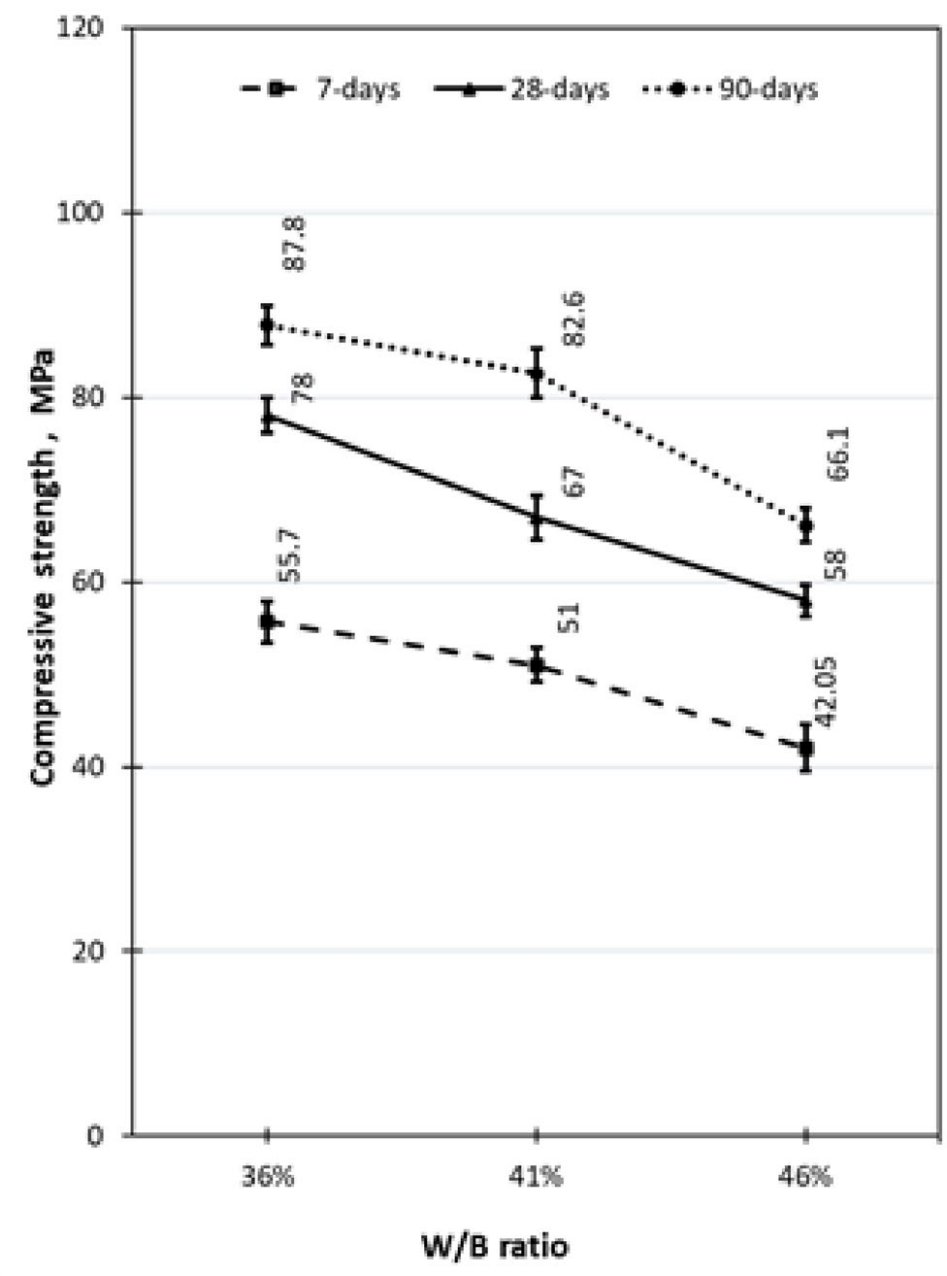
Effect of water–binder ratios on compressive strength.

Figure 8 demonstrates the influence of the W/B ratios on compressive strength for mixtures of M1S1P1, M2S1P2, and M3S1P3 with silica fume and iron powder. As seen in the figure, we can notice after 7 days of curing. It was found that the W/B ratio of 0.36 resulted in a remarkable increase in $f_{c}^{\prime }$ by 9.21% and 32.46% compared to the W/B ratios of 0.41 and 0.46, respectively. It is noted that it increased from 55.7 MPa at age 7 days to 78 MPa and 87.8 MPa after curing for 28 and 90 days, respectively. In a similar vein, after 28 days of curing, the water-to-binder ratio of 0.36 showed significant improvement in compressive strength of roughly 16.4% and 34.48% compared to when the W/B ratios of 0.41 and 0.46, respectively. Also, it increased from 51 MPa at 7 days to 67 MPa and 82.6 MPa after curing for 28 and 90 days, respectively.

Furthermore, at 90 days of curing, the W/B of 0.36 has 6.3% and 32.8% higher compressive strength than water-to-binder ratios of 0.41 and 0.46, respectively. In the same context, it increased from 42.05 MPa at 7 days to 58 MPa and 66.1 MPa at ages 28 and 90 days, respectively. These results are compatible with the substitution of cement for SF, FA, and W/B, as reported by^9,33–35^.

The results presented in Table 7 suggest a difference in $$f_{t} /f_{c}^{\prime }$$ and $$f_{b} /f_{c}^{\prime }$$ of specimens after curing 7 days, 28 days and 90 days with statistical analysis. In Table 7, the $$f_{t} /f_{c}^{\prime }$$ ratios are varied from 11.85, 12.18, and 11.96% at curing 7, 28, and 90 days, with a Mean value equal to 12.00% for the water-to-binder 36%, in other words, *f*_*t*_ = 12.00% $$f_{c}^{\prime }$$$$f_{c} ^{\prime}$$. This result is according to Neville^33^. While it was a Mean value equal to 9.87% at different curing times for the water-to-binder 41%, therefore, the relationship between tensile to compressive strength ratio is equal *f*_*t*_ = 9.87% $$f_{c}^{\prime }$$. On the other side, a water-to-binder ratio of 46% resulted in notable oscillations in the $$f_{t} /f_{c}^{\prime }$$. ratio of curing times. It was 12.13%, 9.83%, and 11.32% after curing for 7, 28, and 90 days, respectively, with a Mean value equal to *f*_*t*_ = 11.09% $$f_{c}^{\prime }$$. This result is confirmed by^30^.

$f_{b} /f_{c}^{\prime }$ relationships is Table 7 with the water-to-binder of sustainable concrete at curing of 7, 28, and 90 days. The $f_{b} /f_{c}^{\prime }$ ratios have a slight change, with mean values of 14.52%, 13.69%, and 13.60% for the water-to-binder ratios of 36%, 41%, and 46%, respectively. Therefore, the relationship between flexural-to-compressive strength ratio is equal *f*_*b*_ = 14.52% $f_{c}^{\prime }$, *f*_*b*_ = 13.69%$f_{c}^{\prime }$, and *f*_*b*_ = 13.60%$f_{c}^{\prime }$ for water-to-blinders of 36%, 41%, and 46%, respectively. These findings agree with the results of^25,35^.

### Impact of (silica fume & fly ash) admixture

**Fig. 9:**
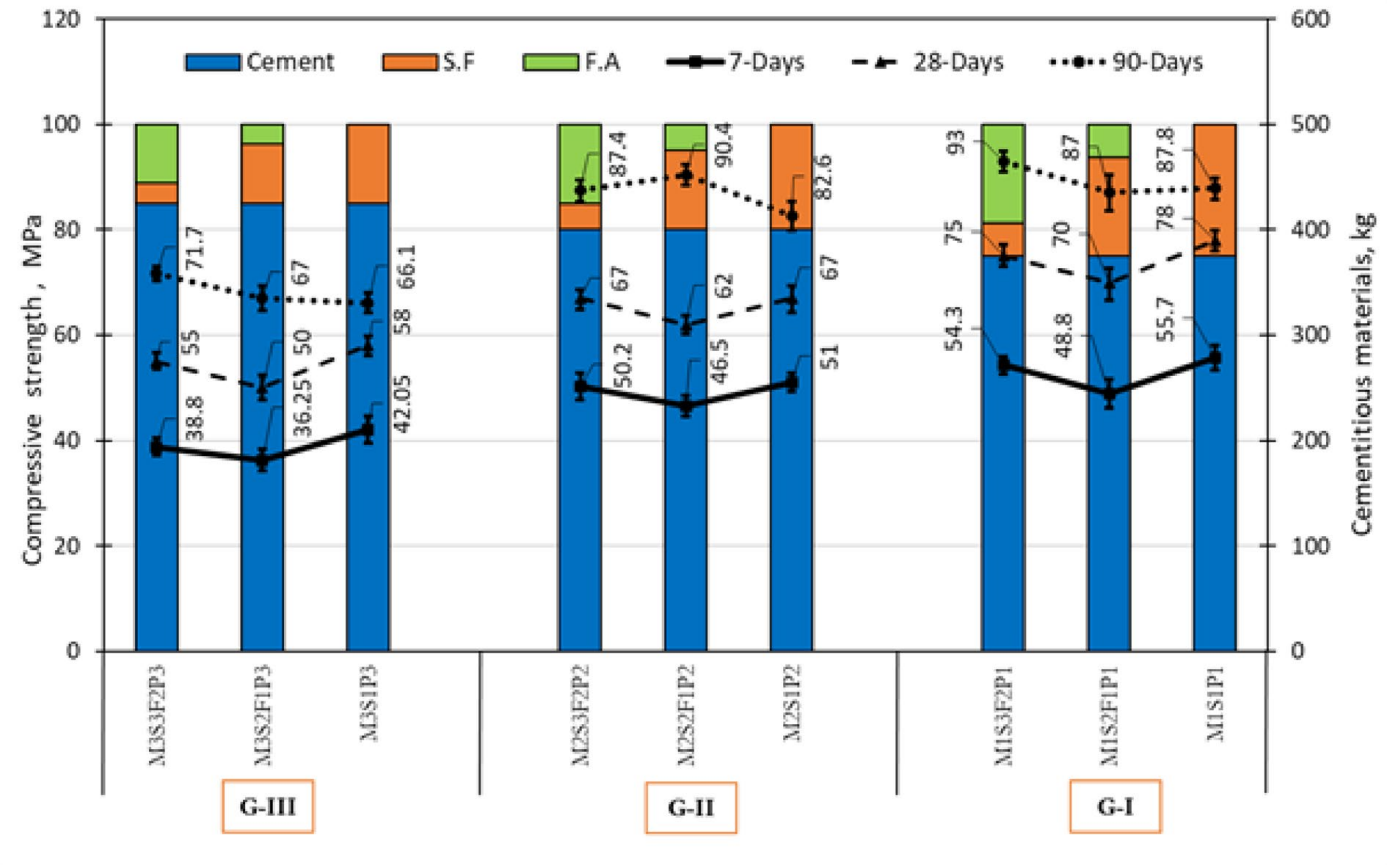
Compressive strength of concretes with different replacement levels of silica fume and fly ash for G-I, G-II and G-III.

Figure 9 shows the effect of the content of mineral admixtures on the compressive strength of concrete for three groups (G-I, G-II, and G-III). Each group is subsequently divided into three mixtures based on replacement level silica fume and fly ash, as shown in Table 1. For group G-I, the compressive strength of mixtures M1S2F1P1 and M1S3F2P1 after 7 days of curing decreased, from 55.7 to 48.8 MPa and 54.3 MPa by 14.14% and 2.57%, respectively, compared to M1S1P1. After 28 days of curing, the findings showed a remarkable decrease in compressive strength by 11.4% for mixture M1S2F1P1 compared to M1S1P1, while $f_{c}^{\prime }$ decreased in for mixture M1S3F2P1 by 3.8%, due to the increasing amount of fly ash to three-quarters of silica fume. Moreover, it was found that after 90 days of curing, the replacement of one-quarter of silica fume with fly ash slightly reduces compressive strength by about 0.9% for mixture M1S2F1P1 compared to M1S1P1, increase in compressive strength for mix M1S3F2P1 by 5.9%, due to the replacement of three-quarters of silica fume with fly ash. In addition, it was a notable improvement in $f_{c}^{\prime }$ for mix M1S2F1P1 and M1S3F2P1, from 70 MPa, and 75 MPa to 87 MPa, and 93 MPa, by about 24.3% and 24%, respectively, after curing for 90 days compared to 28 days.

For group G-II, the compressive strength of mixtures M2S2F1P1 and M2S3F2P3 at 7 days of curing decreased from 51 to 46.5 MPa and 50.2 MPa by about 9.67% and 1.6%, respectively, compared to M2S1P2. After 28 days of curing, the findings showed a notable decrease in compressive strength by 8.06% for mixture M2S2F1P2 compared to M2S1P2, while there was a convergence in the value of compressive strength for mixtures M2S1P2 and M2S3F2P2, due to the increasing amount of fly ash to three-quarters of silica fume. Moreover, it was found that after 90 days of curing, an increase in $f_{c}^{\prime }$ to 90.4 MPa and 87.4 MPa by about 9.44% and 5.8% was observed for mixture M2S2F1P2 and M2S3F2P2, respectively, compared to M2S1P2. Additionally, $f_{c}^{\prime }$ has a significant improvement for mix M2S2F1P2 and M2S3F2P2, from 90.4 MPa and 87.4 MPa to 62 MPa and 67 MPa by about 45.8% and 30.4% higher, respectively, after curing for 90 days compared to 28 days.

The last group, G-III, $f_{c}^{\prime }$ of mixtures M3S2F1P3 and M3S3F2P3 at 7 days of curing, which decreased to 36.25 MPa and 38.8 MPa by 16% and 8.37%, respectively, compared to M3S1P3, which was 42.05 MPa. After 28 days of curing, the results indicated a noteworthy decrease in compressive strength to achieved to 50 MPa and 55 MPa by about 16% and 5.4% for mixture M3S2F1P3 and M3S3F2P3 compared to M3S1P3, which was 58 MPa. Due to the increasing amount of fly ash to three-quarters silica fume. Moreover, it was found that after 90 days of curing, an increase to 67 MPa and 71.7 MPa by1.36% and 8.4% was observed for mixture M3S2F1P2 and M3S3F2P3, respectively, compared to M3S1P3, which was 66.1 MPa. Further, $f_{c}^{\prime }$ value achieved 66.1 MPa, 67 MPa and 71.7 MPa for mix M3S1P3, M3S2F1P3 and M3S3F2P3, by 13.9%, 34% and 30.1%, respectively, after curing for 90 days compared to 28 days.

Table 7 illustrates the $f_{t} /f_{c}^{\prime }$ relationships between the splitting tensile to compressive strength for G-I, G-II, and G-III with varying replacement levels of silica fume and fly ash. the $f_{t} /f_{c}^{\prime }$ ratios were 12.0%, 10.83% and 11.64% for M1S1P1, M1S2F1P1, and M1S3F2P1, respectively. The findings indicate for the first group that slight reduction in the $f_{t} /f_{c}^{\prime }$ ratios with an increase in fly ash to silica fume through (3/4 FA: 1/4SF). On the other side, the second group showed a significant improvement when silica fume was replaced with fly ash, specifically replacing 1/4 of the silica fume with fly ash by 9.96%. Additionally, the $f_{t} /f_{c}^{\prime }$) ratio reached 10.75% in case 3/4 FA: 1/4 SF. While the third group (G-III ) had $f_{t} /f_{c}^{\prime }$ ratios of 11.09%, 10.38%, and 12.16% for M3S1P3, M3S2F1P3, and M3S3F2P3, respectively.

The relationship between the flexural strength-to-compressive strength ratio and various fly ash and silica fume replacement levels for G-I, G-II, and G-III is shown in Table 7. The highest of $f_{b} /f_{c}^{\prime }$ ratios was 15.91%, for M1S3F2P1 in case 3/4 FA: 1/4 SF. It is worth noting that the lowest of $f_{b} /f_{c}^{\prime }$ ratios was 13.62%, for M2S1F1P2 through the replacement of one-quarter of silica fume with fly ash, with a mean value equal to *f*_b_ = 13.51% $f_{c}^{\prime }$ for G-II. On the other hand, the third group showed a significant improvement when silica fume was replaced with three-quarters of fly ash, by 13.77%. Additionally, the lowest of $f_{b} /f_{c}^{\prime }$ ratios was 13.03%, for M3S2F1P3 in case 1/4 FA: 3/4 SF. The results are consistent with the implications of cement substitution with^25^.

### Effect of crushed quartz powder

**Fig. 10:**
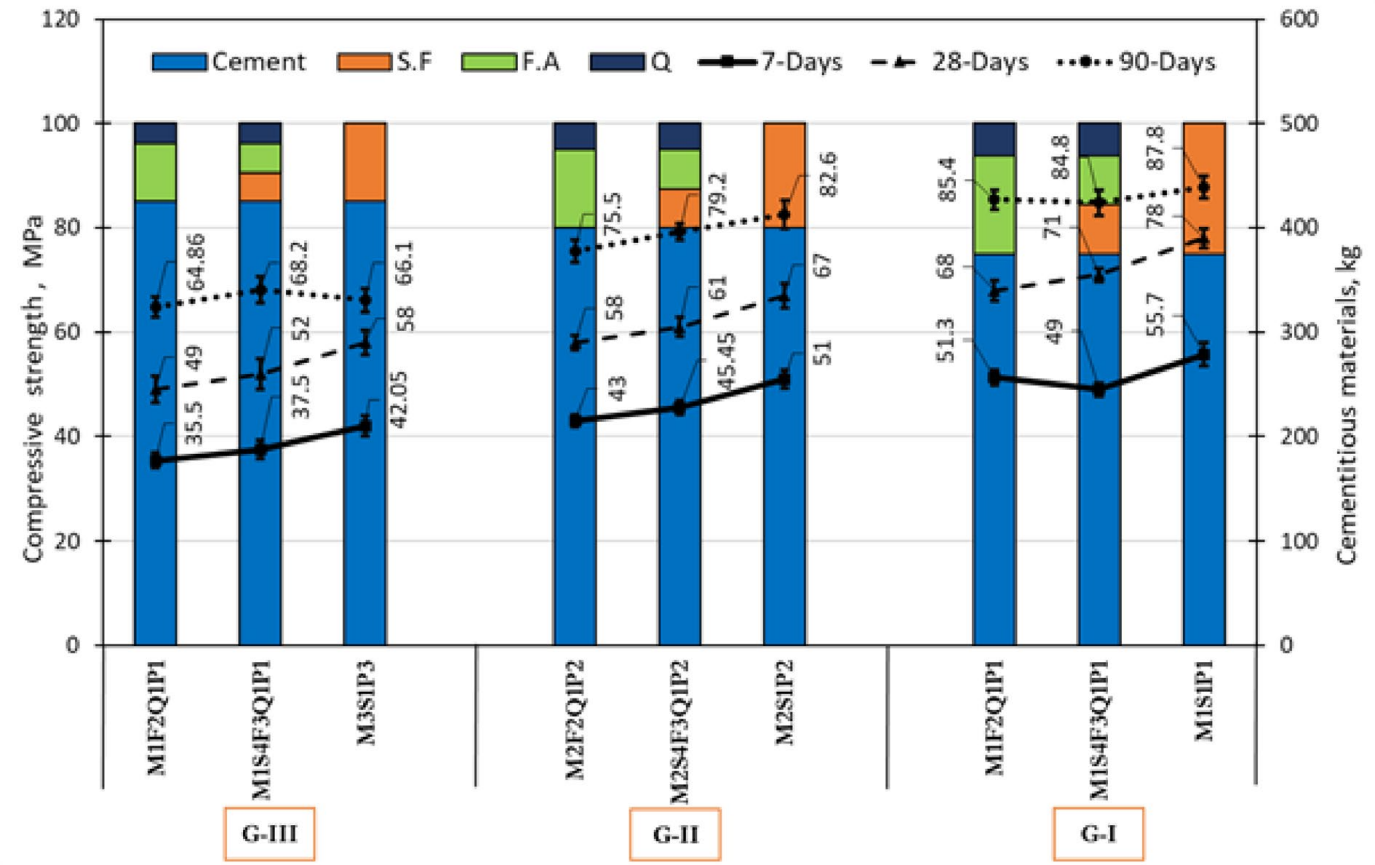
Compressive strength of concretes with different replacement levels of silica fume, fly ash, and quartz powder for G-I, G-II, and G-III.

The effects of crushed quartz powder on concrete’s compressive strength for three groups (GI, GII, and G-III) are shown in Fig. 10. In these groups, half the silica fume was replaced with fly ash in the mixture M1S4F3Q1P1, and all the silica fume was replaced with fly ash in the mixture M1F2Q1P1. Besides, quartz powder is added to two mixes, equivalent to one-quarter of the amount of mineral admixtures. For group G-I, the compressive strength of mixtures M1S4F3Q1P1 and M1F2Q1P1 after 7 days of curing decreased to 49 MPa and 51.3 MPa by about 13.67% and 8.57%, respectively, compared to M1S1P1which was 55.7 MPa. After 28 days of curing, the findings showed a gradual decline in compressive strength to 71 MPa and 68 MPa by about 9.8% and 14.8%, respectively, compared to M1S1P1which was 78 MPa. On the other side, after 90 days of curing, it was a slight decrease in $f_{c}^{\prime }$ for mixture M1S4F3Q1P1 and M1F2Q1P1 to 84.8 MPa and 85.4 MPa by 2.8% and 3.5%, respectively, compared to M1S1P1 which was 87.8 MPa. Further, development in values of $f_{c}^{\prime }$ for mixtures M1S4F3Q1P1 and M1F2Q1P1 reached 84.8 MPa and 85.4 MPa, respectively, after curing for 90 days. In contrast, it was 71 MPa and 68 MPa, respectively, after curing for 28 days.

For group G-II, the compressive strength of mixtures M2S4F3Q1P2 and M2F2Q1P2 at 7 days of curing decreased to 45.45 MPa and 43 MPa by about 12.2% and 18.6%, respectively, compared to M2S1P2 which was 51 MPa. After 28 days of curing, the findings showed a gradual decline in compressive strength by 9.8% and 15.5%, respectively. On the other side, after 90 days of curing, it was a slight decrease in $f_{c}^{\prime }$ for mixture M2S4F3Q1P2 and M2F2Q1P2 to 79.2 MPa and 75.5 MPa by about 4.3% and 9.4%, respectively, compared to M2S1P2 which was 82.6 MPa. For group G-III, *fc* of mixtures M3S4F3Q1P3 and M3F2Q1P3 at 7 days of curing decreased by about 12.1% and 18.45%, respectively, compared to M1S1P1. After 28 days of curing, the results demonstrated a remarkable reduction in $f_{c}^{\prime }$ to 52 MPa and 49 MPa, by about 11.5% and 18.3%, respectively, compared to M3S1P3 which was 58 MPa. On the other side, after 90 days of curing, the $f_{c}^{\prime }$ for mixture M3S4F3Q1P3 increased by about 3.0% to 68.2 MPa, compared to M3S1P3 at 66.1 MPa. In contrast, a notable increase in $f_{c}^{\prime }$ for mixture M3S4F3Q1P3 by about 3.2%, compared to M3S1P3. Further, decrease $f_{c}^{\prime }$ for mixture M3F2Q1P3 was 64.86 MPa, compared to mixture M3S1P3, which reached 66.1 MPa. In contrast, after curing for 90 days, $f_{c}^{\prime }$ values reached 68 MPa and 64.86 MPa, respectively, up from 52 and 49 MPa at curing of 28 days.

The splitting tensile to compressive strength correlations for G-I, G-II, and G-III with different replacement quantities of fly ash, silica fume and quartz powder are shown in Table 7. The $f_{t} /f_{c}^{\prime }$ ratios were 10.50%, 10.04% for M1S4F3Q1P1 and M1F2Q1P1, respectively, compared to 12.0% for M1S1P1. The second batch, with $f_{t} /f_{c}^{\prime }$ ratios of 10.68% and 9.97% for M2S4F3Q1P2 and M2F2Q1P2, respectively, compared to 9.87% for M2S1P2. The last group, with $f_{t} /f_{c}^{\prime }$ percentages of 10.37% and 10.28% for M3S4F3Q1P3 and M3F2Q1P3, respectively, compared to 11.09% for M3S1P3. In general, we notice a decrease in the value of $f_{t} /f_{c}^{\prime }$ when quartz is added.

Table 7 shows the relationships between flexural and compressive strength at various replacement levels of silica fume, fly ash, and quartz powder for G-I, G-II, and G-III. $f_{b} /f_{c}^{\prime }$ ratios were 13.62%, 13.22% for M1S4F3Q1P1 and M1F2Q1P1, respectively, compared to 14.52% for M1S1P1. The second set, with $f_{b} /f_{c}^{\prime }$ ratios of 13.19% and 12.13% for M2S4F3Q1P2 and M2F2Q1P2, respectively, compared to 13.69% for M2S1P2. The last group, with $f_{b} /f_{c}^{\prime }$ percentages of 12.39% and 12.37% for M3S4F3Q1P3 and M3F2Q1P3, respectively, compared to 13.60% for M3S1P3. In general, we notice a decrease in the value of $f_{b} /f_{c}^{\prime }$ when quartz is added.

Chemical composition provides an indication of the possible reactivity of the supplement materials; however, physical and mineralogical characteristics are equally important. Silica fume (SF) is known to contain very fine, non-crystalline, spherical particles with a high specific surface area that react quickly and fill up gaps between other particles^36^. Fly ash (FA) has mostly spherical particles called cenospheres. These cenospheres are mostly amorphous glass containing aluminum and silicon. The activity level of fly ash depends on its fineness and the amount of glass it contains^37^. In contrast, crushed quartz (CQ) is a crystalline material (α-quartz) that acts mainly as an inert micro-filler, helping to refine the microstructure^38,39^. Cooled cast iron (CCI) particles serve as a dense filler because they are composed of metallic iron and do not react. The synergistic effects discussed in this paper arise from the interaction between these reactive phases (SF, FA) and non-reactive phases (CQ, CCI); this mixture affects hydration kinetics, pore structure, and ITZ density. This study was limited to macro-mechanical performance observations for complex blends; further work should include advanced characterization techniques such as X-ray diffraction (XRD), scanning electron microscopy (SEM) with energy-dispersive spectroscopy (EDS), and Fourier-transform infrared spectroscopy (FTIR) applied to both raw materials and hydrated pastes. The results would provide direct microstructural evidence of pozzolanic reactions, filler effects, and ITZ modifications that led to strength development and the synergistic behaviors observed in this study.

### Scan electronic microscopy (SEM)

**Fig. 11:**
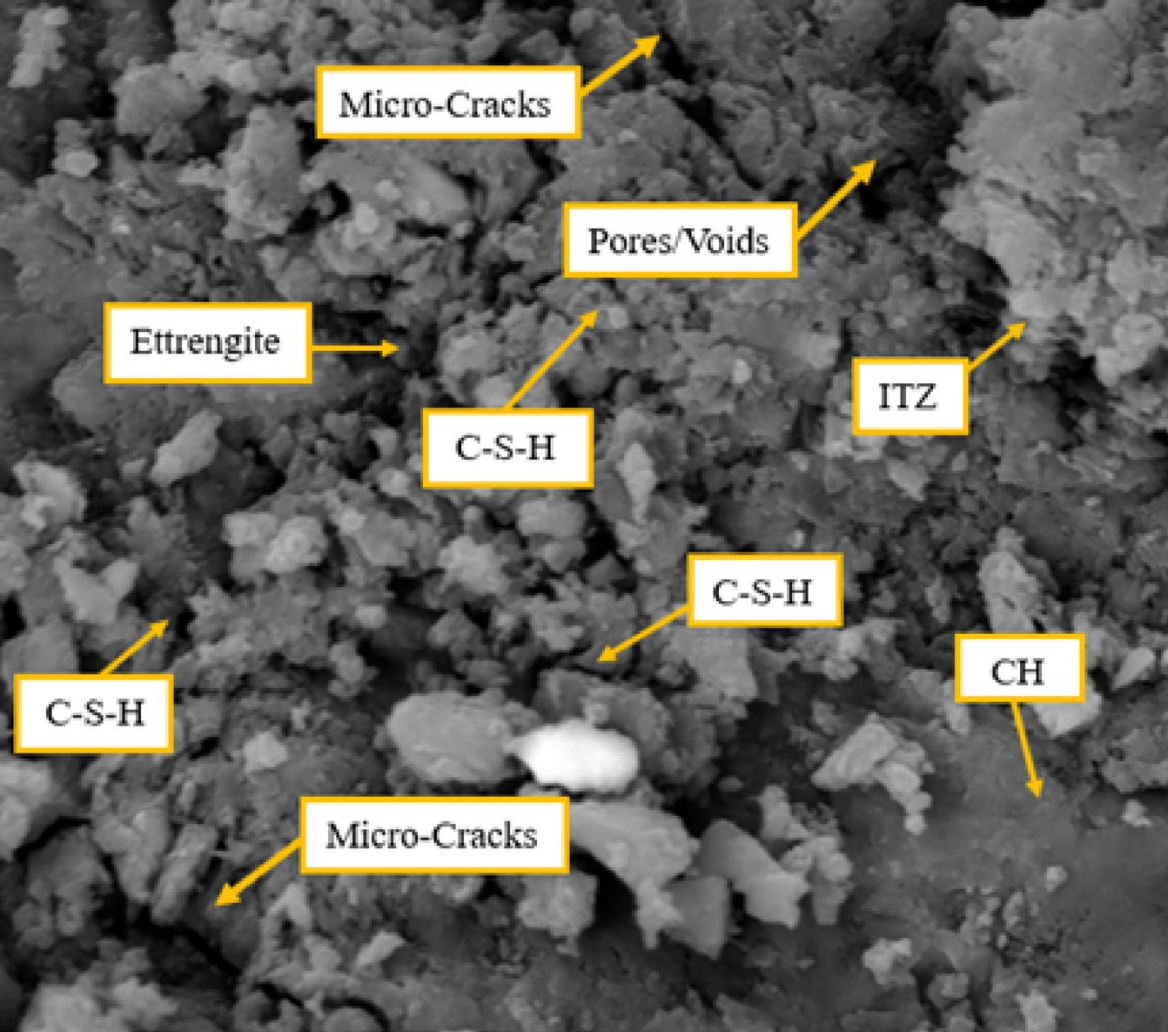
SEM micrographs for mixture M1S2P3 with SF.

The microstructural characteristics of the M1S1P1 concrete, where cement was partially substituted by silica fume, have been captured in the Scanning Electron Microscopy (SEM) micrograph shown in Fig. 11. The SEM image of silica fume mix shows a moderately porous microstructure with visible voids that could be potential weak zones. There is abundant, well-distributed C–S–H, which contributes to the material’s compressive strength. However, the presence of CH indicates an incomplete pozzolanic reaction, which may slightly reduce durability. Ettringite crystals are also seen here as they represent early hydration products. The ITZ appears somewhat less porous, which may help strengthen the bond. In general, the silica fume mix promotes more formation of C–S–H as compared to plain cement or fly ash mix, and keeps the microstructure relatively dense.

**Fig. 12:**
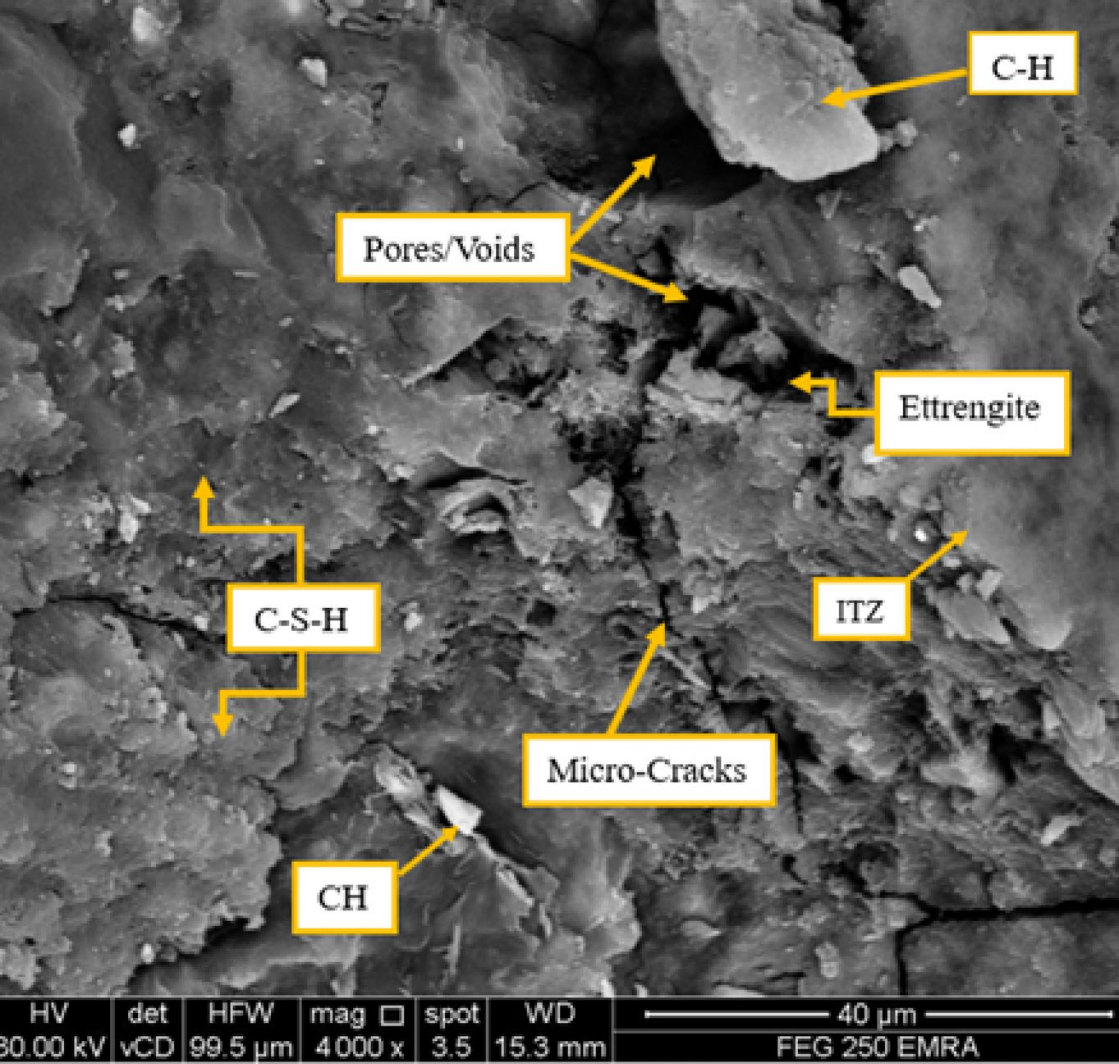
SEM micrographs for mixture M1S2F1P1 with three-quarters of silica fume with Fly Ash.

While mixture M1S2F1P1 was formulated by replacing three-quarters of silica fume with Fly Ash that the microstructural characteristics of the specimen of the SEM micrographs are detailed in Fig. 12. The SEM image of the fly ash (M) shows a microstructure with relatively high porosity. Voids and microcracks are visible, which may indicate weak areas. Calcium Silicate Hydrate (C–S–H), as the binding phase, presents itself in fibrous matrix form, contributing to strength. The presence of Calcium Hydroxide (CH) indicates that the pozzolanic reaction has not been completed; CH is less beneficial. Ettringite crystals can be seen, which means hydration occurs at an early stage. The Interfacial Transition Zone (ITZ) appears weak and less compact; this may contribute to mechanical weakness due to its increased porosity. Although C–S–H formation strengthens the material, visible porosity and microcracks suggest limited improvements in strength and durability.

### Comparison between tensile, flexural, and compressive strengths with the international code

**Fig. 13:**
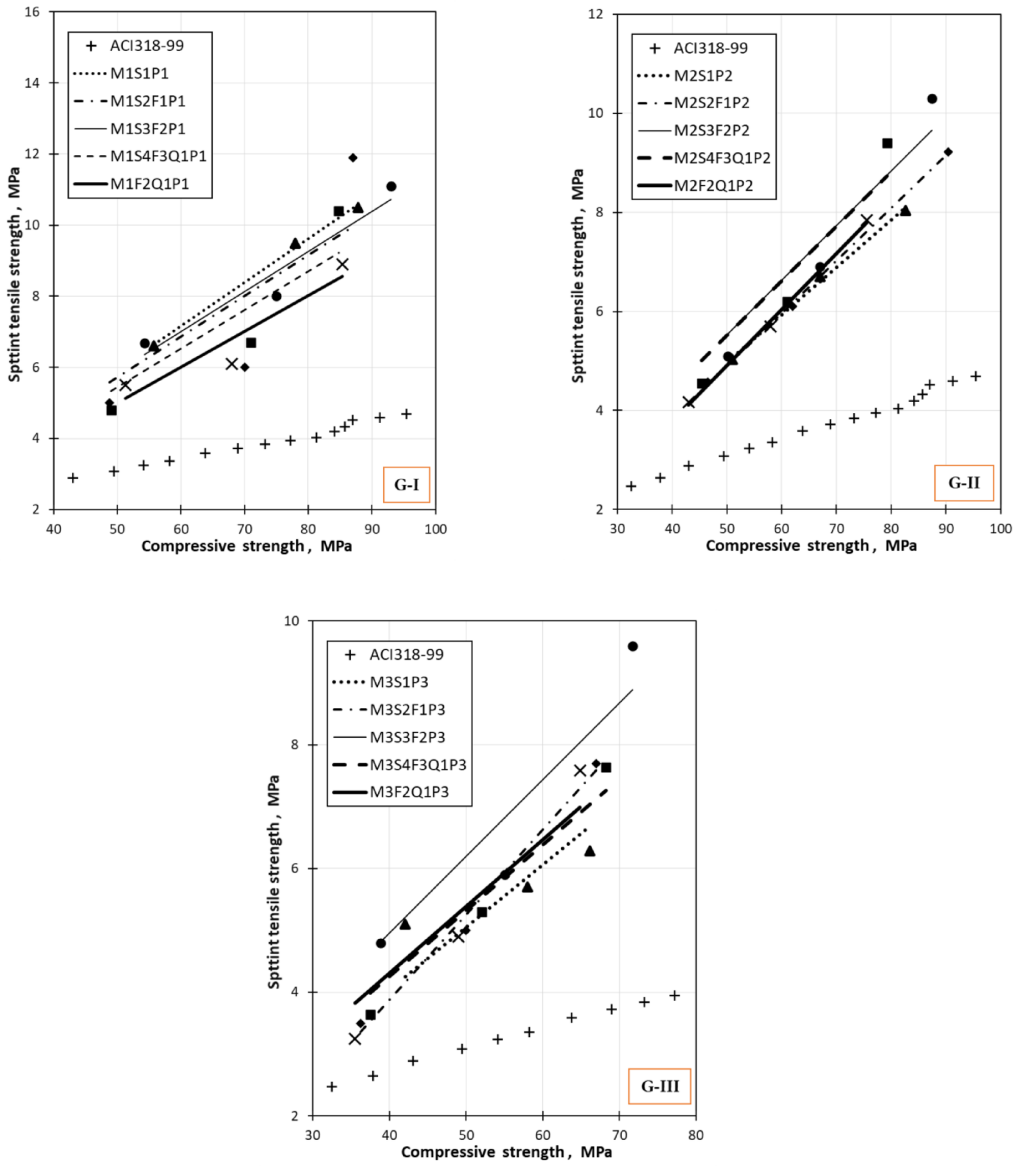
Comparison between tensile and compressive strength of concrete mixes made from industrial wastes with the international code.

Figure 13 shows values of split tensile strength as a function of compressive strength for different mixes in G-I, G-II, and G-III, with comparison to ACI318-99. From Fig. 13, it can be seen that ACI 318 estimates the tensile strength value compared to values for both normal and high-strength concrete. The difference between the calculated and experimental values increases significantly as the concrete strength increases. It can be easily noticed that the current results for different mixtures are higher than ACI318-99, probably due to the presence of varying replacement levels of silica fume, fly ash, and quartz powder.

**Fig. 14:**
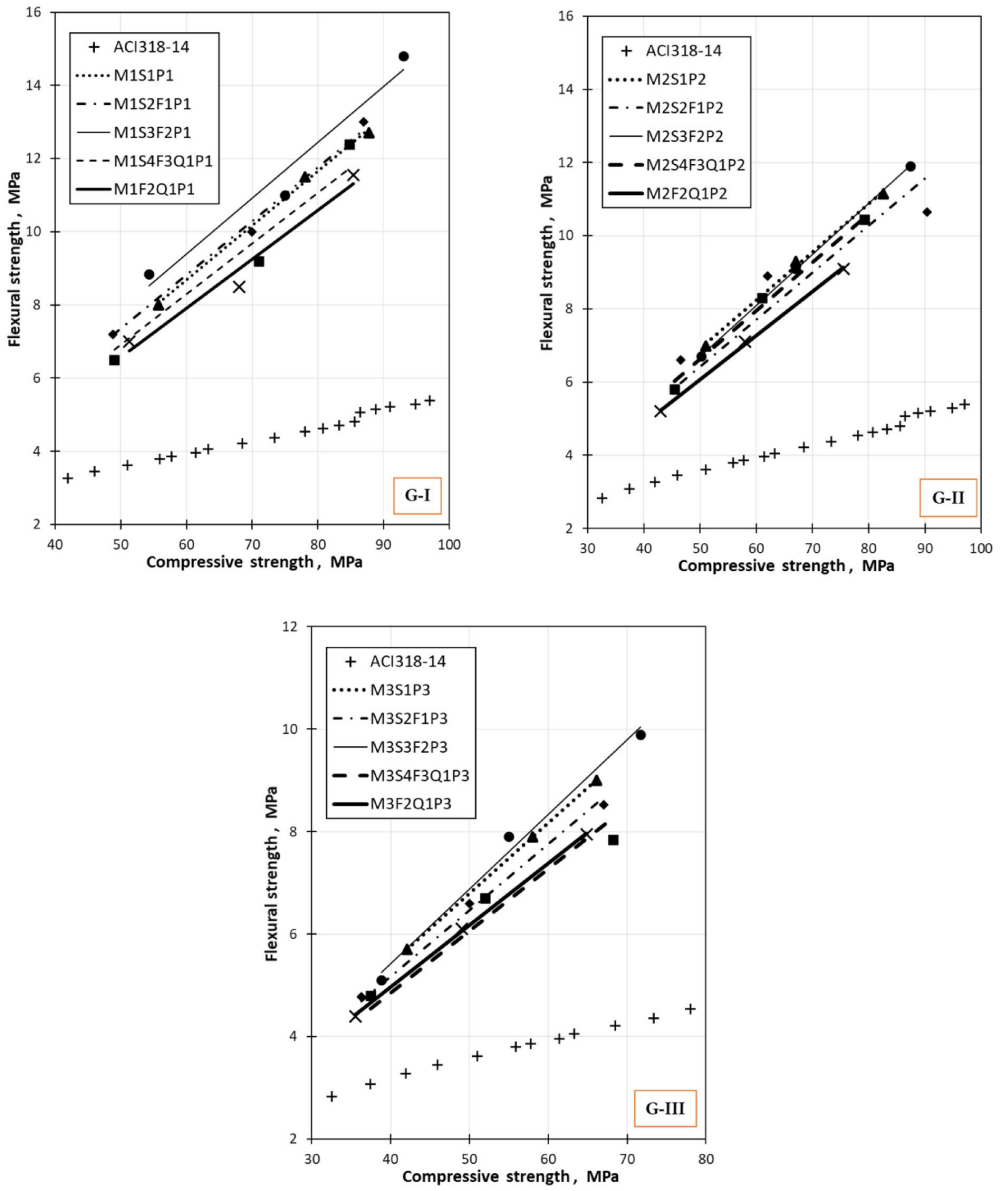
Comparison between flexural and compressive strength of concrete mixes made from industrial wastes with the international code.

Figure 14 shows values of flexural strength as a function of compressive strength for different mixes in G-I, G-II, and G-III, with comparison to ACI318-14. From Fig. 14, it can be seen that ACI 318 estimates flexural strength compared to values for both normal and high-strength concrete. Notably, the current results for different mixtures are higher than ACI318-14, probably due to the presence of varying replacement levels of silica fume, fly ash, and quartz powder.

### Sustainability implications and environmental benefits

Beyond mechanical performance, a principal motivation for this study is reducing concrete’s environmental footprint through industrial waste valorization. The mix designs presented herein directly contribute to this goal by substantially reducing Portland cement demand. For instance, in the M1 series (W/B = 0.36), the total supplementary cementitious material content ranges from 125 to 143.5 kg/m^3^ (Table 1), corresponding to a cement replacement level of 25–28% by mass of the total binder. Using an emission factor widely cited as 0.9 kg CO_2_-eq per kg OPC clinker production [cite e.g., standard reference], this reduction in cement translates into an estimated saving of approximately 101 to 115 kg of CO_2_ equivalent per cubic meter of concrete for these high-strength mixes.

Importantly, mix M1S3F2P1, which replaced three-quarters of the silica fume with fly ash, achieved an excellent 90-day compressive strength of 93 MPa, using only 94 kg/m^3^ fly ash, an industrial by-product with very low embodied energy compared to cement or silica fume. This is indicative that it is possible to maximize the use of lower-impact fly ash, which is more widely available, without jeopardizing long-term performance. Moreover, all mixes successfully incorporated cooled cast iron (CCI) particles, thus valorizing a metal waste stream.

Although a full life cycle assessment falls outside the scope of this mechanical performance study, our quantitative estimates of cement reduction and associated CO_2_ savings are in agreement with findings from dedicated LCA research on sustainable concrete containing similar industrial by-products^3,5,20,40,41^. Results here confirm that optimized blending of SCMs, particularly at lower water–binder ratios, enables the production of high-strength concrete with a significantly improved environmental profile, thus supporting the transition toward circular-economy construction.

## Conclusions

This experimental work was conducted to study the effect of using SF, FA, CCI, and CQ as partial replacements for cement on the hardened properties of concrete, with the water-to-binder ratio (W/B) varied. Based on the results of this work, the following conclusions are summarized.

- The highest compressive, tensile, and flexural strength of concrete containing industrial waste was recorded using the water–binder ratio of be set as 36% after curing for 7, 28, and 90 days.
- The relationship between the splitting tensile to compressive strength ratio ($f_{t} /f_{c}^{\prime }$) was 9.83%, 10.0%, and12.18% for water-to-blinders of 36%, 41%, and 46%, respectively, at curing of 28 days. While $f_{b} /f_{c}^{\prime }$ was 13.62%, 13.88%, and14.74% for water-to-blinders of 36%, 41%, and 46%, respectively, at curing of 28 days.
- The compressive strength has a remarkable increase for mix M1S3F2P1 by 5.9%, due to the replacement of three-quarters of silica fume with fly ash. Additionally, a significant improvement in $f_{c}^{\prime }$ for mixtures M2S2F1P2 and M2S3F2P2, approximately 45.8% and 30.4% higher, respectively, after curing for 90 days compared to 28 days. Further $f_{c}^{\prime }$ has a considerable improvement for mix M3S2F1P3 and M3S3F2P3, around 34% and 30.1%, respectively, after curing for 90 days compared to 28 days.
- The flexural strength remarkably decreased with the variation and inclusion of silica fume (SF), and fly ash (FA) in mixtures after curing for 28 days. While the flexural strength was improved by 16.5%, 6.7%, and 10%, for mixtures of 0.36, 0.41, and 0.46, respectively (when using silica fume 25% and fly ash 75%), after curing for 90 days.
- Adding crushed quartz powder to concrete mixtures achieved acceptable results, which led to a slight decrease in the compressive strength by about 9.3%, 13.4% and 10.9% for mixtures 36%, 41%, and 46%, respectively, after curing for 28 days. On the other hand, it was 8.1%, 13.6%, and 9.5% for mixtures with 36%, 41%, and 46% by volume, respectively, after 90 days of curing.

### Limitations and future research directions

Future research should focus on enhancing the early-age strength of fly ash (FA)-rich concrete mixes, potentially by integrating chemical activators or optimizing curing conditions. Additionally, subsequent studies should employ X-ray diffraction (XRD), scanning electron microscopy with energy-dispersive spectroscopy (SEM–EDS), and Fourier-transform infrared spectroscopy (FTIR) to quantify the correlation between microstructural alterations and mechanical and durability performance. A thorough examination of the effects of curing temperature and prolonged exposure on these blends is necessary, along with a comprehensive life cycle assessment (LCA) to evaluate their environmental impact concerning energy consumption, emissions, and lifetime costs.

## Data Availability

Data is provided within the manuscript.

## List of symbols

$$f_{c}^{\prime }$$: Compression strength
CCI: Cooled cast iron
FA: Fly ash
$$f_{b}$$: Flexural strength
OPC: Ordinary Portland cement
SCM: Scan electronic microscopy
SF: Silica fume
SD: Standard deviation
$f_{t}$: Spitting tensile strength
W/B: Water–binder ratios
QP: Quartz powder
